# Supplementary material for: Structural evidence for Scc4-dependent localization of cohesin loading
Source: eLife. 2015 Jun 3;4:e06057. doi: 10.7554/eLife.06057 (PMC4471937; doi:10.7554/eLife.06057)
Supplement: Supplementary file 1. — Yeast strains used in this study. DOI: http://dx.doi.org/10.7554/eLife.06057.015 [file elife06057s001.docx]

**Supplementary File 1 – Yeast strains used in this study.**

S288c Strains:

| Strain Number | Relevant Genotype | Reference |
| --- | --- | --- |
| BY4741 | *MATa, his3Δ1, leu2Δ0, met15Δ0, ura3Δ0* | - |
| SMH173 | *SCC4(WT)::HISMX* | This work |
| SMH154 | *SCC4(L256K)::HISMX* | This work |
| SMH155 | *SCC4(Y298A;K299D)::HISMX* | This work |
| SMH156 | *SCC4(Y313A)::HISMX* | This work |
| SMH157 | *SCC4(F324A;K327D;K331D)::HISMX* | This work |
| SMH158 | *SCC4(K541A;K542A)::HISMX* | This work |
| SMH191 | *chl4Δ:: KanMX6* | This work |
| SMH172 | *chl4Δ:: KanMX6 SCC4(F324A;K327D;K331D)::HISMX* | This work |
| SMH186 | *SPC110-mCherry::hphMX3* | This work |
| SMH187 | *SPC110-mCherry::hphMX3 chl4Δ:: KanMX6* | This work |
| SMH188 | *SPC110-mCherry::hphMX3 SCC4(F324A;K327D;K331D)::HISMX* | This work |
| SMH189 | *SPC110-mCherry::hphMX3 SCC4(F324A;K327D;K331D)::HISMX, chl4Δ:: KanMX6* | This work |
| SMH283 | *SCC4(L256K;Y298A;K299D;Y313AF324A;K327D;K331D)::HISMX* | This work |
| SMH294 | MATa/α diploid *SCC4::HISMX* | This work |
| SMH296 | MATa/α diploid *chl4Δ:: KanMX6* | This work |
| SMH298 | MATa/α diploid *SCC4(L256K;Y298A;K299D;Y313AF324A;K327D;K331D)::HISMX* |  |
| SMH314 | *SCC4(L256K;Y298A;K299D;Y313AF324A;K327D;K331D)::HISMX chl4Δ:: KanMX6* | This work |
| SMH317 | MATa/α diploid *MTW1-tdTomato::natMX6 SCC2-GFP::HISMX* | This work |
| SMH319 | MATa/α diploid *MTW1-tdTomato::natMX6 SCC2-GFP::HISMX SCC4(L256K;Y298A;K299D;Y313AF324A;K327D;K331D)::HISMX* | This work |

W303 Strains:

| Strain Number | Genotype | Reference |
| --- | --- | --- |
| AM8405 | S*CC2-AID::KanMX, ura3::pADH1-OsTIR1-9MYC::URA3* | This work |
|  |  |  |
| AM1081 | *MATa* *pMET-CDC20 LEU2::tetR-GFP tetO-URA3* (38.4 kb to left of *CEN5*) | Fernius et al., 2013 |
| AM1145 | *MATa SCC1-6HA* | Fernius et al., 2013 |
| AM1176 | *MATa* W303 wild-type | Fernius et al., 2013 |
| AM4643 | *MATa pMET-CDC20* 2.4*CEN4*(2.4 kb right of *CEN4*)::*tetOs tetR-GFP SPC42-tdTomato* | Fernius et al., 2013 |
| AM5329 | *MATa* *pMET-CDC20 LEU2::tetR-GFP tetO*-4.5*CEN6* (4.5 kb right of *CEN6*) *SPB42-tdTomato* | Fernius et al., 2013 |
| AM5533 | *MATa* *pMET-CDC20 LEU2::tetR-GFP tetO*-17.8*CEN5* (17.8 kb left of *CEN5*) *SPB42-tdTomato* | Fernius et al., 2013 |
| AM5545 | *MATa pMET-CDC20 LEU2::tetR-GFP tetO*-12.6*CEN5* (12.6 kb left of *CEN5*) *SPB42-tdTomato* | Fernius et al., 2013 |
| AM6006 | *MATa SCC2-6HIS-3FLAG* | Fernius et al., 2013 |
| AM15307 | *MATa SCC2-6HIS-3FLAG SCC4::HIS3* | This work |
| AM15311 | *MATa SCC2-6HIS-3FLAG scc4m35::HIS3* | This work |
| AM15537 | *MATa SCC1-6HA scc4m35::HIS3* | This work |
| AM15540 | *MATa SCC1-6HA Scc4::HIS3* | This work |
| AM15788 | *MATa pMET-CDC20* *CEN4*(2.4kbR*)::tetOs tetR-GFP SPC42-tdTomato SCC4::HIS3* | This work |
| AM15789 | *MATa pMET-CDC20 CEN4*(2.4kbR)::*tetOs tetR-GFP SPC42-tdTomato scc4m35::HIS3* | This work |
| AM15971 | *MATa pMET-CDC20 LEU2::tetR-GFP tetO*-4.5*CEN6* (4.5 kb right of *CEN6*) *SPB42-tdTomato scc4m35::HIS3* | This work |
| AM15973 | *MATa pMET-CDC20* *LEU2::tetR-GFP tetO*-17.8*CEN5* (17.8 kb left of *CEN5*) *SPB42-tdTomato scc4m35::HIS3* | This work |
| AM16084 | *MATa pMET-CDC20* *LEU2::tetR-GFP tetO-URA3* (38.4 kb to left of *CEN5*) *scc4m35::HIS3* | This work |
| AM16203 | *MATa pMET-CDC20 LEU2::tetR-GFP tetO*-12.6*CEN5* (12.6 kb left of *CEN5*) *SPB42-tdTomato scc4m35::HIS3* | This work |
| AM17882 | *MATa SCC2-6HIS-3FLAG scc4^m7^::HIS3* | This work |
| AM17885 | *MATa SCC1-6HA scc4^m7^::HIS3* | This work |
