# Supplementary material for: Structural evidence for Scc4-dependent localization of cohesin loading
Source: eLife. 2015 Jun 3;4:e06057. doi: 10.7554/eLife.06057 (PMC4471937; doi:10.7554/eLife.06057)
Supplement: Supplementary file 2. — Primers for ChIP-qPCR experiments in this study. DOI: http://dx.doi.org/10.7554/eLife.06057.016 [file elife06057s002.docx]

**Supplementary File 2 – ChIP Primers**

| Primer set | Region (Fig.) | AM Primer | Sequence |
| --- | --- | --- | --- |
| IV-a1 | Arm ChrIV (4B) | 782 | AGATGAAACTCAGGCTACCA |
|  |  | 783 | TGCAACATCGTTAGTTCTTG |
| IV-c2 | *CEN4* (FB) | 794 | CCGAGGCTTTCATAGCTTA |
|  |  | 795 | ACCGGAAGGAAGAATAAGAA |
| IV-p1 | Pericen ChrIV (4B) | 1319 | ATGATTCAATGGATTTAGCC |
|  |  | 1320 | GTCAGTCTTATGCTGTTCCC |
| V-c1 | *CEN5* (4S2A) | 945 | TGAAGGTGAGCTTAAGACAG |
|  |  | 946 | CAACCATGTTCGTAGCTAAA |
| V-a1 | Arm ChrV (4S2A) | 949 | CTACGGTAAATCTGGGTAGG |
|  |  | 950 | TCCACTATCAAGTCACCAGA |
| III-c1 | *CEN3* (4S2A) | 1279 | TGTTGATGGGTTTACAATTT |
|  |  | 1280 | CTTTCAATGATTGCTCTAAATC |
| III-a1 | Arm ChrIII (4S2A) | 1285 | ATGGTACCTAGCTCGTGAAT |
|  |  | 1286 | GGATTTGTCAACTTGGAACT |
